# Multiscale modelling of transport in polymer-based reverse-osmosis/nanofiltration membranes: present and future

**DOI:** 10.1186/s11671-024-04020-w

**Published:** 2024-05-21

**Authors:** Haochen Zhu, Anthony Szymczyk, Aziz Ghoufi

**Affiliations:** 1 State Key Laboratory of Pollution Control and Resources Reuse, Key Laboratory of Yangtze River Water Environment, College of Environmental Science and Engineering, Tongji University, 1239 Siping Rd., Shanghai, 200092 China; 2 CNRS, ISCR (Institut des Sciences Chimiques de Rennes) - UMR 6226, Univ Rennes, 35000 Rennes, France; 3 CNRS, ICMPE (Institut de Chimie et des Matériaux Paris-Est) - UMR 7182, Univ Paris-East Creteil, 94320 Thiais, France; 4 CNRS, IPR (Institut de Physique de Rennes) - UMR 6251, Univ Rennes, 35000 Rennes, France

## Abstract

Nanofiltration (NF) and reverse osmosis (RO) processes are physical separation technologies used to remove contaminants from liquid streams by employing dense polymer-based membranes with nanometric voids that confine fluids at the nanoscale. At this level, physical properties such as solvent and solute permeabilities are intricately linked to molecular interactions. Initially, numerous studies focused on developing macroscopic transport models to gain insights into separation properties at the nanometer scale. However, continuum-based models have limitations in nanoconfined situations that can be overcome by force field molecular simulations. Continuum-based models heavily rely on bulk properties, often neglecting critical factors like liquid structuring, pore geometry, and molecular/chemical specifics. Molecular/mesoscale simulations, while encompassing these details, often face limitations in time and spatial scales. Therefore, achieving a comprehensive understanding of transport requires a synergistic integration of both approaches through a multiscale approach that effectively combines and merges both scales. This review aims to provide a comprehensive overview of the state-of-the-art in multiscale modeling of transport through NF/RO membranes, spanning from the nanoscale to continuum media.

## Introduction

Nanofiltration (NF) and reverse osmosis (RO) are physical separation technologies utilized for eliminating contaminants from liquid streams. In recent years, NF/RO technologies have become increasingly efficient and cost-effective across various industrial applications such as the food industry, biotechnology, wastewater treatment, brackish water and seawater desalination [1]. For example, RO currently accounts for around 70% of the world’s desalination capacity. RO and NF are membrane filtration processes where pressure is applied to a liquid stream, compelling it to pass through a semipermeable membrane to remove dissolved species [2, 3]. These techniques are energy-efficient (as they do not require a phase change for separation), environmentally friendly (requiring little or no addition of chemicals), and are modular and compact compared to distillation or evaporation [4]. NF/RO can effectively remove small molecules, such as pesticides and pharmaceutical compounds. While RO and NF are efficient purification technologies, both necessitate a substantial amount of energy to push liquids through the membranes and their nanosized pores. Consequently, sufficient pressure must be applied to overcome the osmotic pressure, enabling liquid to pass through the membrane. RO represents the most refined of membrane filtration systems, featuring extremely small free volume elements capable of removing particles as small as a few angstroms, while NF offers slightly coarser filtration than RO, capable of removing particles ranging from ⁓ 200 to 1000 g/mol e.g. monovalent salts in desalination such as sodium/chloride ions for RO membranes and polyvalent cations/dissolved organic solute for NF membranes.

The primary driving force for species transport through RO membranes is the gradient of chemical potential across the membrane, where the applied hydraulic pressure plays a crucial role. Along this gradient, liquid molecules move through the membrane, while the transport of solutes like salt ions is largely impeded. TFC-RO membranes are formed through the polymerization of monomers, resulting in dense polyamide layer with nanopores that facilitate nanoseparation and selectivity. Key polymers utilized in the fabrication of reverse osmosis (RO) and nanofiltration (NF) membranes include disordered polyamides, cellulose acetate, cellulose diacetate, cellulose triacetate, and piperazine. Polyamide, a macromolecule containing recurring amide (–CO–NH–) groups, can be found both naturally and synthetically. Cellulose-based polymers are typically prepared through phase inversion.

The structure of a polymer thin film composite membrane comprises a thin selective barrier layer atop a porous support. The support possesses a microporous structure (ultrafiltration membrane) to provide mechanical strength and high water flux, while the barrier layer facilitates ion/solute separation. Commercial RO polyamide thin film composite (TFC) membranes are commonly fabricated through interfacial polymerization (IP), which involves impregnating a porous polymeric support with an aqueous m-phenylenediamine (MPD) solution followed by exposure to an organic trimesoyl chloride (TMC) solution. Let us mention that NF membranes use different types of aqueous phase monomer such as piperazine (PIP).

Another emerging type of membrane consists of ordered polymer films containing nanochannels and nanoporous polymer materials with 3D-interconnected structures formed via the direct cross-linking of bicontinuous cubic lyotropic liquid crystal (LLC) phases formed by reactive LLCs [5]. These materials exhibit significant potential for various transport applications, such as desalination. However, their limited use in industry can be attributed to challenges in controlling the effective (1) size of nanopores, (2) producing high-quality thin films, (3) stability i.e., chemical and mechanical steadiness of the thin films in the long-term; (4) scalability, i.e., difficult to mass produce and (5) high production costs [6].

This review primarily focuses on established disordered membranes, such as polyamide.

Areas for improvement primarily focus on enhancing the transport of liquids across membranes to reduce energy costs [7] and membrane selectivity [8]. A thorough understanding of the physical processes governing transport is essential for better control and design of NF/RO membranes. In both NF and RO processes, fluids are confined at the nanoscale (0.1–0.3 nm for RO and 1–2 nm for NF), exhibiting significantly distinct properties from their bulk counterparts. At this scale, physical properties such as solvent and solute permeabilities are intricately linked to the atomic and molecular interactions [9–12]. The microscopic mechanisms underlying reverse osmosis (RO) and nanofiltration (NF) processes are still not fully understood, and gaining insight into these mechanisms is crucial for the long-term conception and design of membranes with enhanced performance. Currently, due to the lack of understanding regarding the molecular mechanisms governing membrane performance in RO/NF systems, decisions regarding the selection of an appropriate membrane/solvent system for a specific application rely on costly and time-consuming screening experiments. Furthermore, it was well known that the RO/NF performance are limited by a selectivity/permeability trade-off relationship The most typical and straightforward approach to enhancing the selectivity of nanochannels is to reduce their pore size, but that comes at the expense of permeability. To overcome this tradeoff, some researchers have proposed to modify the pore profile and chemistry of membrane protein channels while maintaining a similar pore size [8]. An attractive strategy to overcome water permeability trade-off relies on the introduction of additional molecular transportation pathways in “low flux” polymers [8], as carbon nanotube, aquaporins or artificial water channels, in order to increase the membrane permeance while maintaining high solute rejection. However, their optimization and improvement require a thorough understanding of the physical processes occurring at the nanoscale, as pores and additional transportation pathways are nanometric.

Numerous studies have initially concentrated on developing continuum-based transport models [13, 14] aimed at providing insights into the separation properties at the nanometer scale. However, continuum-based models possess limitations: (i) they do not take into account the discrete nature of matter and its impact on the properties of the confined liquid, and therefore they use either the properties of the bulk-phase liquid, or the properties under confinement are fitting parameters for these models, and (ii) they tend to be more descriptive than predictive due to their dependence on fitting parameters (some of which are likely to be dependent on the composition of the liquid, preventing “universal” calibration of the model from fits made on a series of experiments conducted with a “reference” liquid.). This limitation restricts their applicability, for instance, in the generalization and extension to multicomponent mixtures. Nanoconfined systems are recognized for their unique characteristics, including structural arrangements and phase transitions (such as freezing, melting, capillary condensation, and mesomorphic transitions). Continuum descriptions of hydrodynamics and interactions at this scale are prone to breakdown. Molecular simulations of fluids confined in nanopores offer distinctive opportunities to link macroscopic properties to a microscopic description of physical phenomena observed in nanoconfined phases.

More recently, substantial efforts have been invested in developing polymer-based membranes using in-silico approaches [15–17] and studying solute/solvent/polymer interactions [18, 19]. However, comparatively less attention has been devoted to comprehending the transport mechanisms through realistic polymer membranes [20, 21]. Several molecular dynamics simulation studies have focused on modeling fluid transport through various pore models (such as carbon nanotubes [22], spherical pores [23], silica pores [24], etc.).

To sum up, continuum-based models heavily rely on bulk properties, neglecting crucial factors such as liquid structuring, pore geometry, and molecular/chemical specifics. Molecular/mesoscale simulations, while encompassing these details, often face limitations in time and spatial scales. Consequently, achieving a comprehensive understanding of transport requires a synergistic integration of both approaches through a multiscale approach that combines and merges both scales effectively. This review aims thus to provide a comprehensive overview of the state-of-the-art in multiscale modeling of transport through NF/RO membranes, spanning from the nanoscale to continuum media.

## Continuum-based (CB) modelling

### Models based on thermodynamics of irreversible processes

A first approach to describing transport across a membrane is phenomenological. It derives directly from the thermodynamics of irreversible processes, which states that when different fluxes generated by different forces coexist in the same system, the flux ***J***_***i***_ is not only related to its conjugate force ***X***_***i***_, but results from all the forces involved in the overall process [25]. The resulting models treat the membrane as a black box separating two compartments. Their major advantage lies in the fact that no information about the membrane structure is required, making them highly generalized and suitable for both porous and dense membranes.

Building on the pioneering work of Onsager [26], Staverman showed that all transport phenomena across a membrane could be described, in an *n*-component system, by *n*(*n* + 1)/2 phenomenological coefficients [27].

It has been recently established, that for a system of a solvent and a neutral solute, the phenomenological relationships between the volume and the molar flux of solute as function of three transport coefficients, the membrane hydraulic permeability, the solute permeability and the reflection coefficient [28]. However, since the flux equations are based on the linear theory of the thermodynamics of irreversible processes, they are only valid for small values of the forces acting on the system. As this condition is unlikely to be met in NF/RO, where large pressure and concentration gradients can develop across the membrane, Spiegler and Kedem developed an approach consisting in fictitiously cutting the membrane into a numerus slabs, each slice separating two fictitious solution elements (of sufficiently close virtual concentrations to ensure locally linear variation of the fluxes with the acting forces) in thermodynamic equilibrium with the faces of the membrane slice under consideration [29]. The so-developed equations take on a local character, as do the transport coefficients [30]. By integrating these local equations over the membrane thickness (Δ*x*), they established the following equation linking the solute rejection (*R*) to the volume flux through the membrane (*J*_*v*_), the solute permeability (*P*_*s*_) and the reflection coefficient (*σ*_*s*_):

1 $$R=\frac{{\sigma }_{s}(1-\mathit{exp}(-{J}_{v}(1-{\sigma }_{s})\Delta x/{P}_{S}))}{1-{\sigma }_{s}\mathit{exp}(-{J}_{v}(1-{\sigma }_{s})\Delta x/{P}_{S})}$$

The Spiegler-Kedem model was applied in several cases and is still often used. Its main weakness lies in the procedure for integrating local flow equations, which assumes that local transport coefficients are independent of the virtual concentrations (and thus independent of the local position in the membrane). While this hypothesis is correct for neutral membranes, it is more questionable for charged membranes separating partially or fully ionized solutes [31].

### Solution-diffusion model

The solution-diffusion model, first proposed by Lonsdale et al. [32], originates from a simplification of the equations of the thermodynamics of irreversible processes. It assumes no coupling between the solvent and solute fluxes in the membrane, which results in reflection coefficient being assumed to be equal to 1 for all solutes [33]. Another assumption in the solution-diffusion model is that the pressure within the membrane is uniform and equal to the pressure in the feed solution. Consequently, the chemical potential gradient across the membrane (i.e. the driving force) reduces to a concentration gradient and the molar flux of a solute *i* (***j***_***i***_) across the membrane is expressed as follows:

2 $${{\varvec{j}}}_{i}=-{P}_{i}\frac{d{c}_{i}}{dx}$$

where *ci* is the concentration of *i* in the virtual solution and *P*_*i*_ is the permeability of *i* (assumed to be constant throughout the membrane), which is defined in the solution-diffusion model as the product of its local diffusion coefficient and sorption coefficient.

The transfer mechanism of a solute *i* across the membrane is considered to result from a first step in which the compound dissolves in the membrane phase (sorption), followed by a second step in which it diffuses through the membrane down a concentration gradient before finally desorbing and reaching the low-pressure compartment [34]. Selectivity between species arises from their different solubilities in the membrane phase (leading to different sorption coefficients) and their different diffusion rates through the membrane. An extension of the original solution-diffusion, called solution-diffusion-electromigration model, has been developed by Yaroshchuk et al. to account for the electrically coupled transport of ions in electrolyte mixtures [35].

The assumption that there is no coupling between solvent and solute fluxes (and therefore no convective transport in the membrane) makes the solution-diffusion model suitable for dense membranes (i.e. without pores). It is the most widely accepted continuum-based model for reverse osmosis (as well as for gas permeation and pervaporation) although a recent study has called into question the validity of the solution-diffusion mechanism in reverse osmosis membranes [36].

### Nanopore models

A more mechanistic approach is based on the use of the extended Nernst–Planck equation [37], which considers transport across the membrane as resulting from (i) convection generated by the pressure gradient and the coupling between solute and solvent fluxes, (ii) diffusion caused by the concentration gradient induced by the pressure gradient applied across a selective membrane [36], and (iii) electromigration (for charged species only) under the action of the electric field developing spontaneously across the membrane to ensure electroneutrality of the solution transferred into the low-pressure compartment:

3 $${{\varvec{j}}}_{i}=-{D}_{i}\nabla {c}_{i}-\frac{F{z}_{i}{D}_{i}{c}_{i}}{RT}\nabla \psi +{c}_{i}{\varvec{u}}$$

In Eq. (3), *R* is the ideal gas constant, *T* the temperature, *F* the Faraday constant, *c*_*i*_, *z*_*i*_, and *D*_*i*_ are the local concentration, the charge number and the diffusion coefficient of the ion *i*, respectively, **u** is the fluid velocity and *ψ* is the electrical potential that are given by Navier–Stokes and Poisson equations, respectively:

4 $$-\nabla P+\eta {\nabla }^{2}{\varvec{u}}-F{\sum }_{i}{c}_{i}{z}_{i}\nabla \psi =\rho {\varvec{u}}\nabla {\varvec{u}}$$

5 $${\nabla }^{2}\psi =-\frac{F}{{\varepsilon }_{0}{\varepsilon }_{r}}{\sum }_{i}{c}_{i}{z}_{i}$$

where *P* is the hydrostatic pressure, *ε*_0_ the vacuum permittivity, and *ε*_r_ and *η* the solution dielectric constant and viscosity, respectively.

**Fig. 1:**
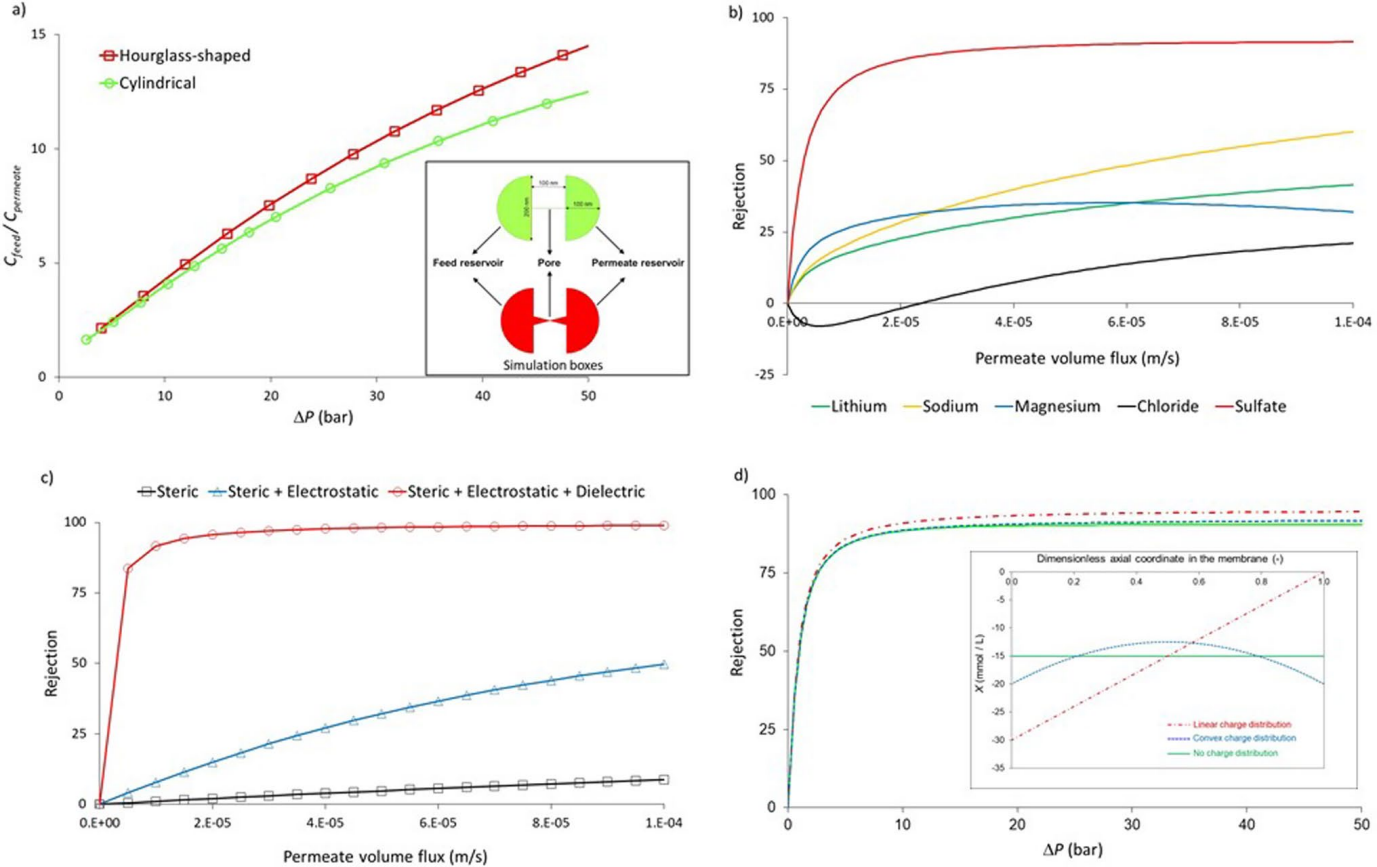
**a** PNP-NS model [59]. Purification factor of cylindrical (diameter: 1.2 nm) and hourglass-shaped (base diameter: 1.6 nm, tip diameter: 0.8 nm) nanopores vs. applied pressure difference. Surface charge density: − 2 mC/m^2^; Nanopore length: 100 nm; Solution: 0.001 KCl. **b** SEDE model [41]. Rejection of an electrolyte mixture (feed concentration: 5 meq/L for each species) by a nanoporous membrane (pore diameter: 2 nm; thickness to porosity ratio: 5 µm; membrane volume charge density: − 100 mmol/L) vs. permeate volume flux. **c** SEDE model [60]. Rejection of a centimolar NaCl solution by a nanoporous membrane (pore diameter: 1 nm; thickness to porosity ratio: 1 µm) vs. permeate volume flux for various exclusion mechanism: steric hindrance, steric hindrance + Donnan (electrostatic) exclusion (membrane volume charge density:—50 mmol/L), steric + Donnan (electrostatic) exclusion (membrane volume charge density:—50 mmol/L) + dielectric exclusion (dielectric constant of the solution inside (outside) pores: 40 (80)). **d** SEDE model with spatial charge density distributio [61, 62]. Rejection of a millimolar KCl solution by nanopores (diameter: 4 nm, length: 2 µm) with various distributions of the volume charge density (see inset) vs. applied pressure difference. Average volume charge density: − 15 mmol/L for all distributions

Equations (3)–(5) are the governing equations of the so-called Poisson/Nernst-Planck/Navier–Stokes (PNP–NS) theory. It provides a sound framework for the study of ion transport through a charged porous membrane. However, even though most (nano)pore models describe the membrane as a bundle of straight, parallel cylindrical or conical pores (reducing the system to a 2D problem), solving the system of nonlinear Eqs. (3)–(5) remains cumbersome and the PNP-NS framework (or related approaches) has been little used for the study of ion rejection in pressure-driven membrane processes (Fig. 1a) [37–42].

An alternative approach combining the extended Nernst-Planck equation with the fixed charge theory was developed by Schlögl [43] and Dresner [44, 45]. In this approach, the exclusion of solutes by the membrane is ruled by (Donnan) partitioning equations (assuming local thermodynamic equilibrium at the membrane/solution interfaces) while the solute transport inside the membrane is described by means of the extended Nernst–Planck equation coupled with a local electroneutrality condition:

6 $${\sum }_{i}{z}_{i}{c}_{i}+X=0$$

where *X* is the volume charge density of the membrane.

By doing away with the Poisson equation, replaced by the local electroneutrality condition, this approach allows a substantial decrease of computational efforts as well as a straightforward extension of the model to electrolyte mixtures (Fig. 1b).

Originally, the notion of the pore was absent from the approach developed by Schlögl and Dresner. It was later introduced by Bowen et al. who added a size exclusion factor (explicitly taking into account the pore size of the membrane) in the partitioning equations at interfaces. This approach led to the development of the DPSM model which has been very widely used in NF [43–45]. Applying the approach of Schlögl and Dresner to a (nano)porous membrane amounts to implicitly neglecting the radial variations of the electrostatic potential in the membrane pores, and leads to 1D transport models. Such a uniform potential approximation, where the local ion concentrations and electric potential are defined as radially averaged quantities, has been shown to be reliable for weakly charged membranes when the Debye screening length in the solution is much larger than the pore size [46, 47]. When applied to membrane transport, the extended Nernst–Planck equation is commonly modified by friction factors or hydrodynamics factors depending on the extent of solute–membrane friction [48–51].

In addition to considerably reducing the computational efforts compared to the PNP-NS theory, an advantage of approximate models based on the uniform potential approach is the possibility of straightforwardly incorporating different exclusion mechanisms, such as steric hindrance and dielectric effects (Fig. 1c) [52–56], as well as membrane/solute affinity [57, 58], via the partitioning equations at the membrane/solution interfaces. It is also simple to account for a heterogeneous distribution of charged groups in the membrane (Fig. 1d) by defining a local volume charge density in the membrane via equation (6) [59, 61, 62].

## Force field molecular (FFM) simulations

NF/RO membranes are constructed with nano-porosity, and gaining insight into fluid transport requires a nanometric perspective. However, the limited knowledge regarding fluid transport in confined media significantly hampers our ability to model interactions between liquids and membranes, and more broadly, our comprehension of the behavior of confined fluids. Simulations at a molecular scale can offer microscopic insights that are otherwise experimentally inaccessible or challenging to attain, thus elucidating the underlying physics from the ground up. In recent years, molecular simulations have seen increasing use in investigating solvent and solute transport through polymer membrane models. This section aims to provide a critical review of atomistic simulation for modeling transport properties at the microscopic scale.

### NF/RO membranes construction

**Fig. 2:**
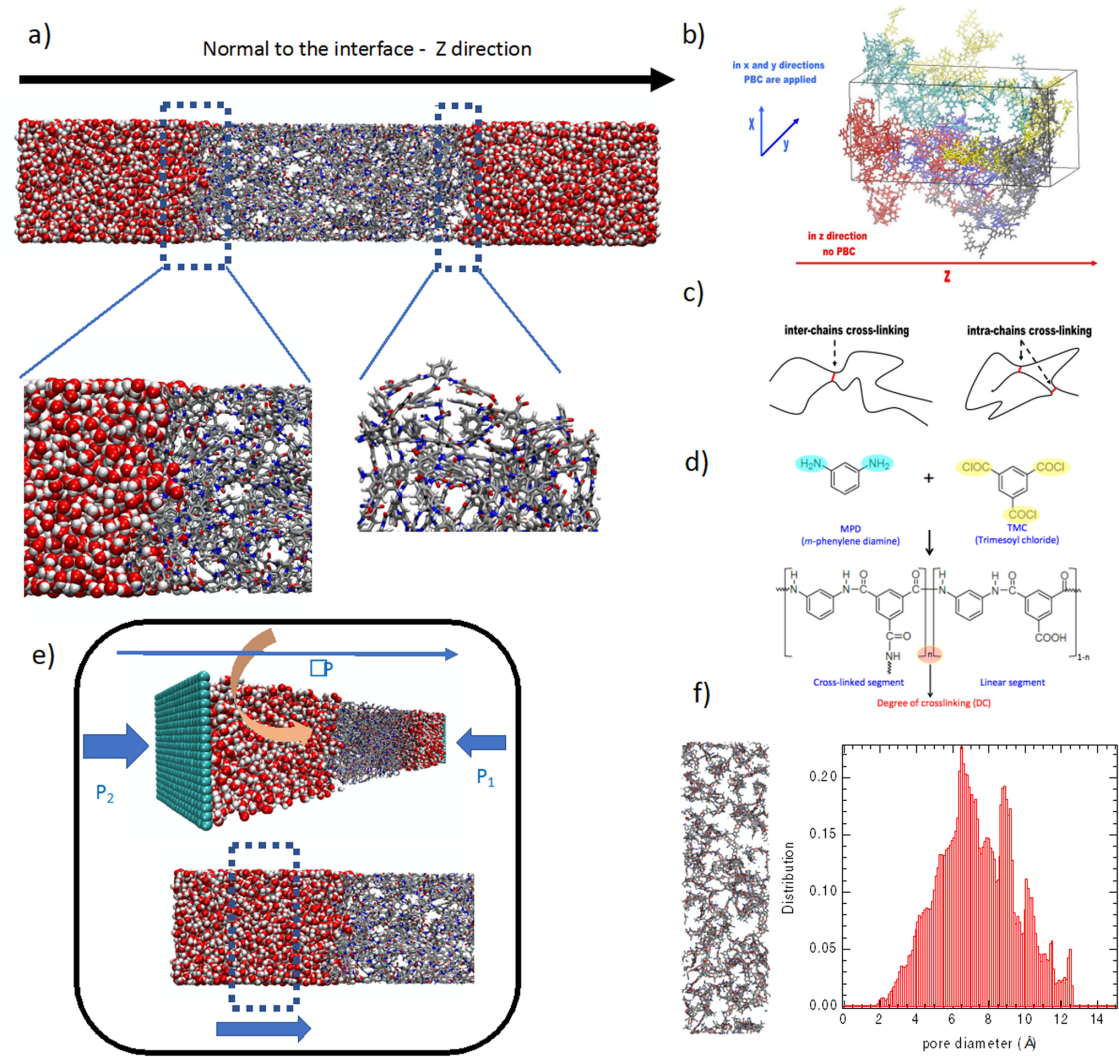
**a** Illustration of a polymer (polyamide)/water interface along its normal. Red, gray, white and blue colors represent oxygen, carbon, hydrogen and nitrogen atoms respectively. **b** bulk of linear polymer chains inserted by means of Material Studio using the Theodorou and Seuter algorithms [50]. Each chain is represented by a different color. Periodic boundary conditions (PBC) are also indicated according to direction. **c** Illustration of inter- and intra-crosslinking between two polymer chains. **d** Illustration of reactive sites on two monomers MPD and TMC leading to in-silico polymerization. **e** illustration of pressure driven simulation using graphitic walls [70, 71] (straight blue arrows), force on liquid molecules [66–68] (curved pink arrow) and from a central slab [72] (dashed box). **f** Pore diameter distribution of the polyamide membrane associated with a snapshot of the membrane porosity with a similar color code to that in part **a**)

The two most popular approaches used to construct a polymeric membrane are, in chronological order, (i) building a polymer chain and randomly inserting it into a simulation box [63–66], and (ii) in-silico polymerization from a monomer bath [16, 17, 67]. Once the polymer has been constructed, its relaxation must be carried out through a series of controlled simulations at high temperatures and pressures. Modeling fluid transport requires consideration of transport through the interface between the polymer and the liquid, as highlighted in Fig. 2a, implying the use of an anisotropic system. Furthermore, as is often the case, nanofiltration or reverse-osmosis membranes (thin polymeric layer) are strongly cross-linked, meaning that chemical bridges exist between linear chains.

#### Construction from a polymer chain

In this case, the membrane is constructed by considering N chains, each composed of m atoms of x monomers. This is achieved using a Monte Carlo construction method based on the statistical bias developed by Theodorou & Seuter [68], which is well-integrated in BIOVA Material Studio [69]. Initially, a monodisperse uncross-linked polymer is obtained (Fig. 2b). Prior to relaxing the polymer, monomer molecules are introduced into the simulation box to create a cross-linked polymer (Fig. 2c). To do so, a distance criterion based on the calculation of the pair correlation function from a monomer bath is performed to allow for the creation or deletion of chemical bonds. Care must be taken in this procedure, for instance to prevent newly created bonds from intersecting with an aromatic ring [65, 67], and a strategy is devised to assign partial charges after each creation or deletion. Subsequently, the empty cross-linked polymer (dehydrated polymer) is equilibrated using for instance the method based on the scheme [70]; 7 cycles of 3 molecular dynamics simulations are performed: (1) in canonical ensemble at 600 K; (2) in NVT with a temperature of 300 K; and (3) in isothermal-isobaric ensemble at 300 K, where N is the number of atoms and V corresponds to the volume. The pressure in the isothermal-isobaric simulations is progressively increased from the first to the third cycle, up to a maximal pressure of 50 kbar, and then is reduced until it reaches the final value of 1 bar.

#### In-silico polymerization

Alternatively, the polymer can be build using the numerical polymerization method developed by Roux et al. [67], Zhang et al. [17], Nagaoka et al. [16], and Abbott et al. [15]. This methodology has been demonstrated to be suitable for constructing amorphous ladder backbone polymers, including PIM-1 among others [15]. The process is described as follows: (i) an initial random configuration of N monomers is equilibrated. (ii) Initially, the monomers are distributed in an orthorhombic cell with specific dimensions, as such an anisotropic system, with one dimension larger than the other two, is necessary for studying transport through the interface (Fig. 2a). The equilibration is performed through successive Molecular Dynamics runs (MD) in the canonical, isobaric-isothermal, and anisotropic isobaric-isothermal (pressure is kept constant in the normal direction to the polymer interface) ensembles, at 300 K, and at 1 bar. (iii) In a second step, the box length is extended along the z-axis by adding two empty boxes. This step ensures that the monomer density is high enough to get a suitable initial configuration to begin the polymerization in terms of monomer contact, while also being low enough to obtain long chains with no overlaps. (iv) The polymerization process consists of a chemical reaction between two chemical groups (see Fig. 2d where colored ellipsoids highlight the chemical centers of two m-phenylene diamine and trimesoyl chloride monomers to form a polyamide membrane) if they are close enough together (need to define a critical distance [15–18, 67, 71]) (v) finally, an additional step after polymerization involved "capping" the resulting polymers to prevent the presence of high-energy sites from causing any unphysical artifacts. The polymerization resulted in a polydisperse mixture. The primary advantage of this approach lies in its ability to construct cross-linked polymers without the need for additional insertion of monomer molecules, as well as the possibility to investigate different monomer ratios physically.

#### Force field (FF)

In recent decades, numerous force fields have been developed to model polymers, with the most commonly used ones being PCFF [72], CCVF [73], Dreiding [74], AMBER [75], COMPASS [76], and OPLS-AA [77]. These force fields are frequently employed to model various polymers such as polyamide [78, 79], Nafion [80], polydimethylsiloxane [71], polysulfo [66], among others, yielding reliable results in terms of structure, density, and liquid permeability. The choice of the force field (FF), which defines the physical interactions between particles, is crucial for obtaining realistic behavior and quantitative data. However, special attention must be given to the combination of polymer and liquid force fields in terms of the cross Lennard–Jones parameters. Indeed, some are 12–6 while others employ 9–6 to model van der Waals interactions with different Lennard–jones mixing rules. It is necessary to test two or three force fields to ensure that the obtained results are not dependent on the specific FF chosen. In a recent study, the OPLS-AA force field was employed to describe both liquids and polymers, as well as their interactions [19]. The OPLS-AA parameters were optimized to accurately represent experimental properties of liquids, such as density and heat of vaporization. The free energy of hydration of organic molecules using the TIP4P water model [81] was also a crucial component of the optimization process. This makes it a strong candidate for studying membranes in the presence of water (utilizing the TIP4P model) and other organic components. Regarding the water model, recent research has highlighted that the TIP4P model is likely the most suitable for reproducing the structure and dynamics of confined water within a polymer [66, 78–80].

### Filling the polymer from equilibrium molecular dynamics simulation (EMD)

It is now well established that nanofiltration performance, including transport properties and rejection rates, is closely linked to molecular interactions between fluids and polymers. Over the past two decades, this method has provided intricate insights into membrane-solute interactions, solute distribution within the membrane, and the dynamic behavior of solutes. To facilitate these analyses, solvation/hydration of the polymer matrix must be achieved. Several possibilities can be considered: (i) Random insertion of solvent/solute molecules, taking into account experimental densities [63, 64]. (ii) Physical filling using two liquid reservoirs and applying pressure normal to the interface. This can be achieved by employing an anisotropic barostat (NP_n_T) in a periodic box [82] and rescaling the positions of all atoms. Another option is to use one or two walls on which a force along the z-axis is applied to replicate the applied pressure [78–80]. In both methods, the liquid must diffuse through the polymer surface, necessitating the construction of an anisotropic membrane as depicted in Fig. 2a, where the liquid can diffuse through the polymer surface and fill it. The construction of the polymer is similar to that provided in Sects. 3.1.1 and 3.1.2, except that the polymer is confined in one direction (often z) between two liquid reservoirs. (iii) Another method could involve filling through osmotic molecular dynamics simulations [83], while fixing the chemical potential of the liquid. The main advantage of this approach is the absence of liquid reservoirs, which significantly reduces computational time since slow diffusion through the interface is then avoided. Furthermore, this method allows for the consideration of three-dimensional swelling of the polymer, unlike the method discussed in (ii), which only allows for swelling normal to the liquid/polymer interface. Additionally, methods involving liquid reservoirs require a sufficiently large polymer thickness (beyond 100 Å) to prevent interactions between the two interfaces [81].

### Non-equilibrium molecular dynamics simulations

This section delves into the utilization of Non-Equilibrium Molecular Dynamics (NEMD) simulations for examining pressure-driven flow and transport properties of liquids through NF and RO membranes. To simulate the pressure difference in molecular dynamics simulations, an external constant force, **F**_**z**_, is applied along the z-direction to all solvent and solute molecules, as illustrated in Fig. 2e. This force is exclusively applied to the centre of mass of molecules to avoid introducing spurious rotational dynamics [84]. For the purpose of this discussion, it is necessary to keep the centre of mass of the polymer membrane at a fixed in position. The pressure difference across the membrane is connected to F_z_ from ΔP = P_1_ − P_2_ = nF_z_/A [84–86]. As a result, the solvent flux through the polymer membrane can be readily quantified by counting the solvent molecules passing through the membrane during the simulation. An alternate approach consists on the introduction of a non-uniform force to consider the different geometries of the reservoirs and that of a nanotube [87]. Another methodology demonstrating the feasibility of conducting NEMD simulations by incorporating two movable piston [88, 89]. This approach has been successfully employed to generate a pressure-driven flow of water across polymers [78]. More recently, Bocquet et al. extended the method originally developed by Goldsmith and Martens to apply pressure drops through a nanopore [90]. The authors then define a slab of a specified width inside the liquid reservoir, referred to as the control slab and positioned at the centre of the liquid reservoir, in which a set of molecular forces is applied to all atoms in relation to the pressure gradient (see Fig. 2e).

### Mesoscale simulations

The main challenge of atomistic simulation lies in the extensive computational time required to sample low spatial and temporal scales, given the size of the polymer chain. Consequently, transport properties are calculated without the relaxation of polymer chains, leading to the absence of a crucial physical aspect: the interplay between transport properties and polymer swelling. The polyamide matrix indeed displays rotational relaxations, evidenced by partial flips of the MPD cycles with two amplitudes (15° and 40) [91]. It was thus shown a correlation between pore size of the polymer membrane and the water diffusion which is of order of 1 nm. In line with the amplitude of polymer rotational relaxation of MPD cycles suggesting a coupling between polymer relaxation and translational dynamics of confined water in the polymer matrix. Often, polymeric membranes are crosslinked and lack flexibility, which justifies the use of atomistic simulation, as the focus is on liquid transport and polymer/liquid interactions. However, when the polymer is not crosslinked, this consideration becomes significant. In such cases, it becomes relevant to employ mesoscale simulation, which operates at an intermediary scale capable of encompassing extensive spatial (micrometer) and temporal (microsecond) ranges. However, in contrast to atomistic models, universal force fields are lacking. Despite the efforts of the MARTINI group [92], which has developed a simple generic coarse-grained potential, alternative solutions are needed. These may include the use of dissipative particle dynamics (DPD) [93] or many-body dissipative particle dynamics [94, 95], capable of considering both attractive and repulsive contributions. While this approach is promising, it requires a systematic parametrization of force fields tailored to the specific system [96]. Furthermore, force field parametrization is dependent on the statistical ensemble, potentially necessitating additional development to obtain force field parameters for non-equilibrium simulations aimed at calculating transport properties. Very recently, Dequidt et al. have developed an elegant method known as the Bayesian method, capable of providing mesoscopic force field parameters from a tabulated force field [97]. However, similar to DPD, this method is dependent on the statistical ensemble, and further developments will be required to implement mesoscopic non-equilibrium molecular dynamics simulations.

While mesoscopic simulations are well-suited for capturing physical properties at long time and spatial scales, we remain unconvinced that coarse-grained descriptions are relevant in the case of NF/RO membranes, as the free volume elements can be smaller than 5 Å (see Fig. 2f). Moreover, understanding molecular interactions, such as hydrogen bonds, is fundamental for rationalizing transport properties. However, an interesting perspective could be to use coarse-grained simulations to construct cross-linked polymers and then rebuild an atomistic model from the coarse-grained one. Sutton et al. have recently demonstrated that this approach is highly pertinent for mimicking interfacial polymerization computationally [98].

### Atomistic and mesoscopic software

While standard molecular dynamics simulations can be performed using free software such as DL_POLY [99], CHARMM [100], GROMACS [101], LAMMPS [102], and NAMD [103], none of them are capable of conducting osmotic MD simulations without new implementations. BIOVA Material Studio [69] is a fee-based code capable of building confined and bulk un-crosslinked polymers using the method developed by Theodorou and Seuter [68]. CHARMM [100], Polymatic [15], and PKlinks [17] are only capable of performing in-silico polymerization based on heuristic distance criteria. It should be noted that LAMMPS is likely the most versatile code, where numerous polymer force fields such as CVFF and PCFF are implemented. Additionally, Polymatic is coupled with LAMMPS, making it the most suitable tool. It is also possible to develop a custom code in order to manage the creation and deletion of chemical bonds, as well as the updating of force field files according to your specific needs.

## Multiscale strategy

**Fig. 3:**
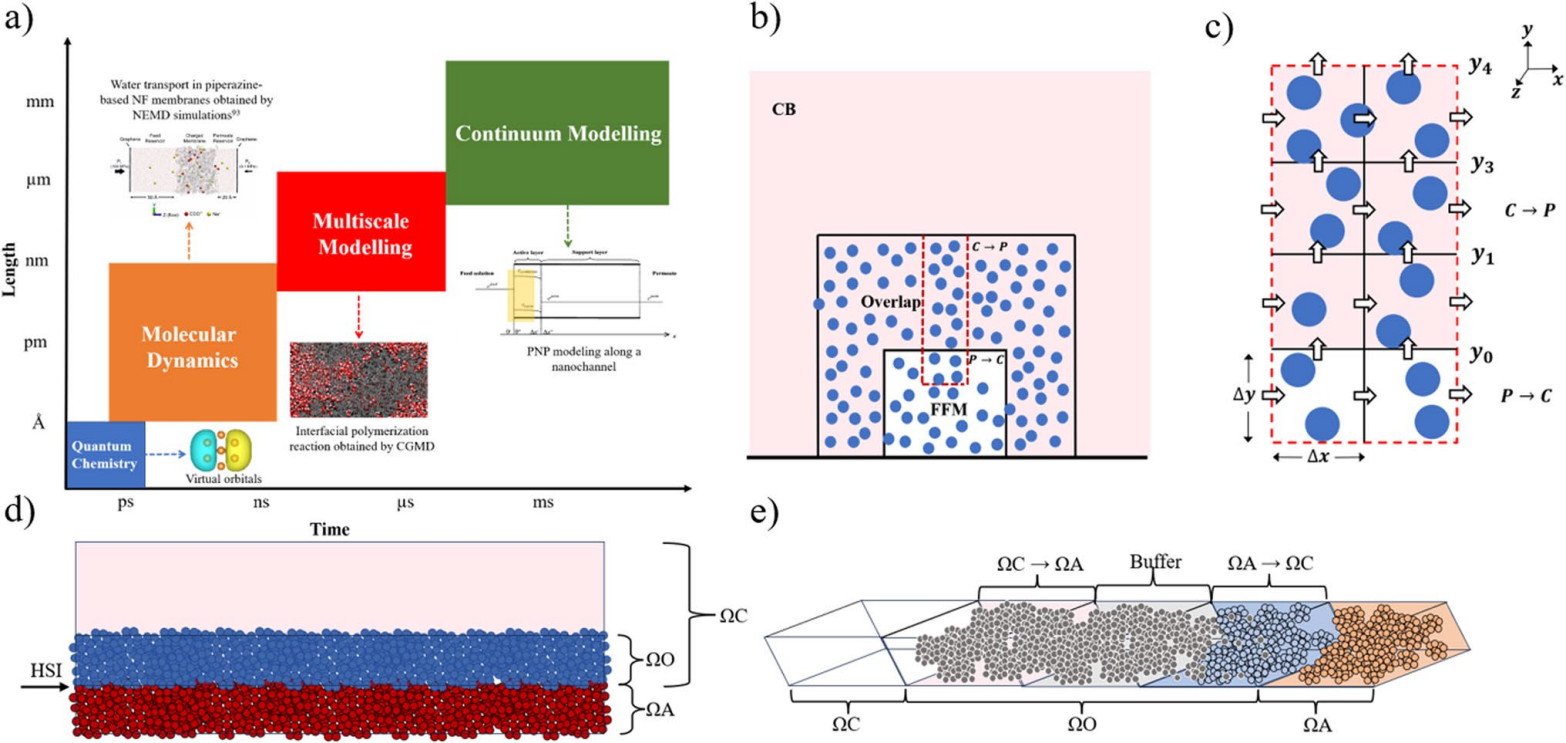
**a** Simulation techniques span quantum to continuum scales, offering diverse approaches for investigating the dynamics of systems across varying time and length scales. Molecular simulations, for instance, enable the determination of diffusion coefficients for ions and water molecules within a membrane matrix. The inset figure of water transport in piperazine-based NF membranes obtained by NEMD simulation is reprinted from Ref. [113] with permission. **b** and **c** The continuum-based (CB) models is used in the shadowed region and the atomistic description is used in the dotted region. In C → P, continuum solutions provide boundary conditions for MD simulations and in P → C atomistic solutions provide boundary conditions for continuum simulations. **d** Nanochannel flow simulated in O’Connell and Thompson [114]. Ω_C_ continuum subdomain, Ω_A_ atomistic subdomain, Ω_O_ overlap region, HSI hybrid solution interface. **e** Representation of domain decomposition in flux-exchange-based HAC models [115]

Connecting the realms of CB modelling and FFM simulations presents a formidable challenge in multiscale modeling due to profound disparities in spatial and temporal scales. FFM simulations excel in unraveling molecular-level intricacies, unveiling individual molecule behaviors [104–106], interactions [107–109], and dynamics [110–112] on the nanometer scale. In the context of polymeric membranes, FFM simulations scrutinize the swift motions of solvents and solutes within nanoscale channels, capturing events occurring from picosecond to nanosecond timescales. In contrast, CB models describe systems as continuous media and handling variables like concentration, pressure, and velocity over much larger scales. Simulation techniques span quantum to continuum scales, offering diverse approaches for investigating the dynamics of systems across varying time and length scales, as shown in Fig. 3a. In membrane transport, CB models predict overall fluid flow and solute transport across the entire membrane structure, encompassing higher timescales, that can go well beyond the scale of a second. This profound mismatch in spatial and temporal scales necessitates a mapping approach—a bridge—capable of translating intricate microscopic data from FFM simulations into parameters that integrate seamlessly within CB models. This mapping is indispensable for achieving an unified understanding of transport phenomena across scales, enhancing predictive accuracy, and leveraging the strengths of both microscopic and macroscopic realms for real-world applications like water purification and materials design. By navigating this scale integration challenge, scientists and engineers will unlock the full potential of multiscale modeling, addressing pressing global challenges across diverse fields.

### Mapping CB methods for mass transport to FFM simulations

The integration of FFM simulations into continuum-based (CB) models has been crucial in advancing the understanding and efficiency of polymer-based reverse osmosis/nanofiltration membranes [116, 117]. FFM simulation provides a detailed view of atomic and molecular interactions, capturing the nuances of individual molecular behaviors (density, viscosity, dielectric permittivity…) and the forces they experience, which is particularly vital in understanding the molecular-level interactions and selectivity of these membranes [118–120]. On the other hand, CB models deal with averaged properties, which is essential for understanding the overall performance and efficiency of the membranes [121–123].

The first step in this integration is the process of parameterization, as elucidated by Kacar [124] and Sengul [125]. This involves converting intricate molecular data from FFM simulations into representative or averaged values suitable for CB models. For polymer-based reverse-osmosis/nanofiltration membranes, this could mean parameterizing diffusivity values of various solutes and solvents to better understand and predict the membrane’s performance. Correlation techniques, highlighted by Zhu [126] and Anderson [127], are also vital in establishing relationships between the microscopic and macroscopic properties. Figure 3b and Fig. 3c illustrate a typical model of multi-scale hybrid simulations. In regions that are homogeneous and have small velocity gradients (shaded region), continuum equations are solved. The key issue is how to couple these very different descriptions of fluids at the MD–continuum interface. Atomistic and continuum descriptions of dense fluids was first made by O’Connell and Thompson, who extended the length scales accessible to molecular dynamics (MD) simulations by coupling it to a continuum Navier–Stokes (NS)-based computation [114]. Particles are governed by Newton's equations of motion. This scheme focused on ensuring the continuity of thermodynamic and transport properties in hybrid solution interface (HSI). As depicted in Fig. 3d, the atomistic subdomain (Ω_A_) is permitted to intersect with the continuum region (Ω_C_) at the interface. Within the overlap region (Ω_O_), it is essential for both the continuum and atomistic descriptions to maintain validity and consistency. The limitations of this model are that the hybrid solution interface (HSI) was parallel to the flow and did not cope with mass transfer across the interface. Hadjiconstantinou and Patera further developed an improved model which was able to deal with mass flow across the HSI and allowed the decoupling of timescales between the evolution of the continuum and the MD solutions [115].

O’Connell and Thompson incorporated the radial distribution function g(r) into hybrid atomistic–continuum (HAC) model to calculate the forces normal to the interface (${\Gamma }_{\mathrm{\Omega O}}$) between Ω_O_ and Ω_C_ and developed a new model that accounts for the local structure of the fluid, the formula is as follows [117].

7 $${F}_{m}\left({r}_{w}\right)=-2\pi {\rho }_{n}\underset{z={r}_{w}}{\overset{{r}_{c}}{\int }}\underset{x=0}{\overset{\sqrt{{r}_{c}^{2}-{z}^{2}}}{\int }}g\left(r\right)\frac{\partial {U}_{12-6}\left(r\right)}{\partial r}\frac{z}{r}xdxdz$$

8 $${U}_{m}\left({r}_{w}\right)=2\pi {\rho }_{n}\underset{z={r}_{w}}{\overset{{r}_{c}}{\int }}\underset{x=0}{\overset{\sqrt{{r}_{c}^{2}-{z}^{2}}}{\int }}g\left(r\right){U}_{12-6}\left(r\right)xdxdz$$

where $r=\sqrt{{x}^{2}-{z}^{2}}$, ${r}_{w}$ is the distance to ${\Gamma }_{\mathrm{\Omega O}}$, ${F}_{m}$ is force components normal to ${\Gamma }_{\mathrm{\Omega O}}$, and ${U}_{m}$ is the potential energy. Wang and He, following the Langevin equation, introduced an external force into the MD equations of the particles to ensure velocity continuity. The modified MD equations were given as follows [116]:

9 $$\frac{{d}^{2}{x}_{i}}{d{t}^{2}}=\frac{{F}_{i}}{{m}_{i}}+\xi \left({u}_{J}-\frac{1}{{N}_{J}}\sum_{i=1}^{{N}_{J}}\frac{d{x}_{i}}{dt}\right)$$

The model presented by Flekkoy couples the atomistic and continuum domains (Ω_A_ and Ω_C_) exclusively through direct flux exchange. The one-dimensional diagram is shown in Fig. 3e, Ω_A_ and Ω_C_ overlap at Ω_O_ [115].

These techniques can be used to correlate variations in molecular density with macroscopic pressure behaviors, enhancing the predictive capabilities of CB models for membrane performance. Furthermore, the coarse-graining method offers a bridge between the molecular and macroscopic scales, allowing for a simplification that retains the essential behaviors of the system [128–130]. Recent advancements in this area have shown that coarse-graining can be effectively used to model complex systems like polymer-based membranes, capturing their unique characteristics and behaviors [131–133].

However, the integration of FFM simulations into CB models for polymer-based membranes is computationally intensive and requires advanced algorithms and high-performance computing, as pointed out by Ando [134]. It is also crucial to ensure that the mapped parameters maintain their physical relevance to prevent the generation of inaccurate CB models [135–137]. Despite these challenges, the rewards of integrating FFM simulations outputs into CB models are significant. As computational power increases and algorithms become more sophisticated, this integration promises to lead to more accurate, predictive models for polymer-based reverse-osmosis/nanofiltration membranes, revolutionizing our understanding and optimization of these crucial technologies in water treatment and separation processes.

Recent studies by Brown [138] and Sadeghi [139] have further emphasized the importance of this integration, especially in the fields of material science and bioengineering, showcasing its potential to drive innovations and optimizations in the design and operation of polymer-based reverse-osmosis/nanofiltration membranes. The convergence of FFM simulations and CB models holds the promise of unraveling the complex interactions at play in these membranes, paving the way for enhanced performance, selectivity, and efficiency, which are crucial for addressing global challenges related to water scarcity and quality.

### Hybrid FFM/CB method: how to manage the FFM/CB interface or information exchange between FFM and CB

#### Establishing an effective interface and ensuring data consistency

**Fig. 4:**
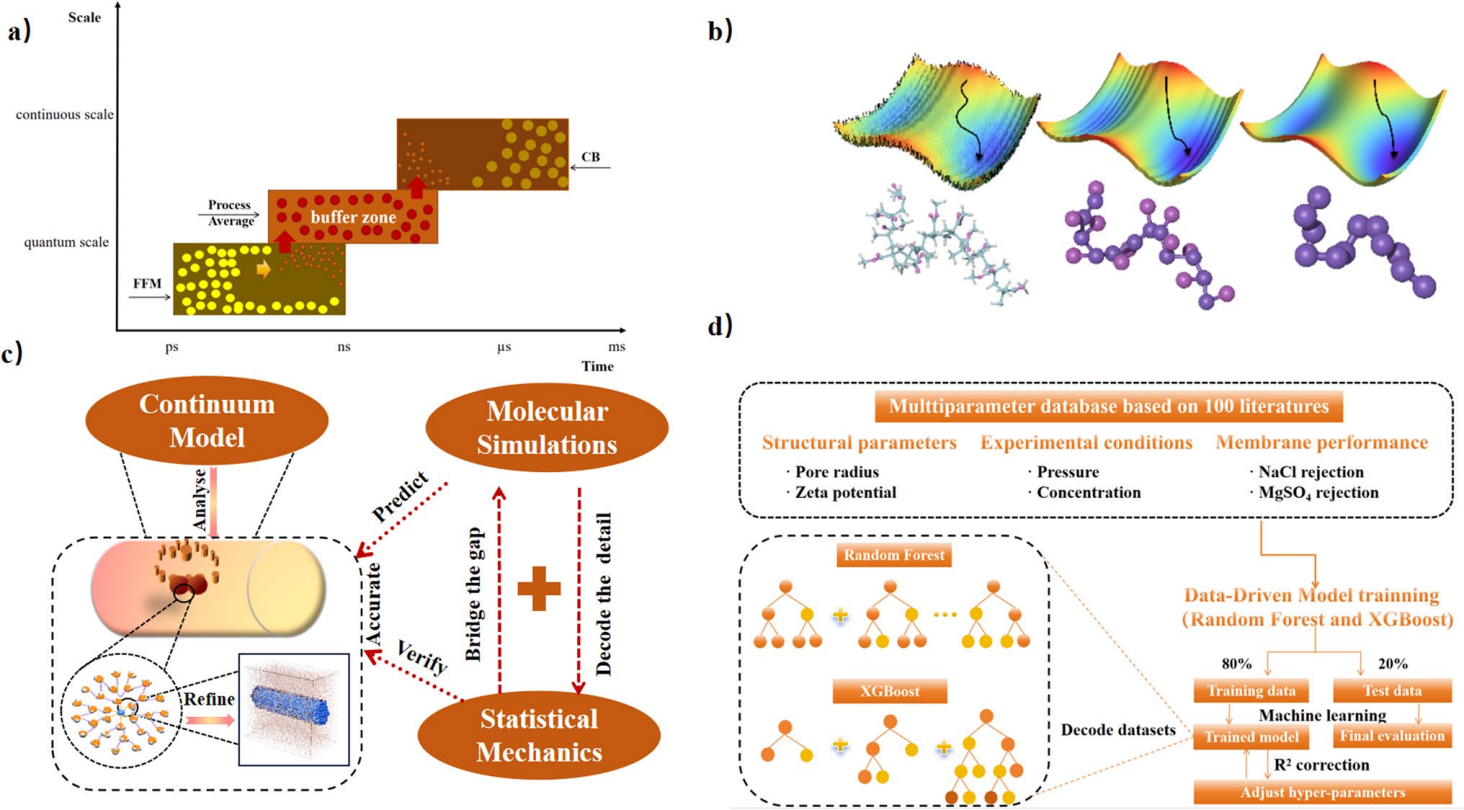
**a** Establish a buffer which processes, averages and transfer data from the FFM simulation to the CB model promotes seamless data transfer between both scales. **b** Data process using coarse-graining techniques. Figure reprinted from Ref. [141] with permission. **c** Continuum model-based analysis and complementary insights from molecular simulations, complemented by integrated validation of hydrodynamic models, enabling more thorough detailed analysis. **d** Machine learning schematic using two boosting tree ensemble models (Random Forest and XGBoost) to explore the correlation between structural parameters, experimental conditions, and performance of NF membranes

The successful integration of FFM simulations with CB models for polymer-based NF/RO membranes necessitate a meticulously designed interface to ensure efficient information flow between the two scales, which will help us build a fundamental understanding of the mechanisms of separation and transport at the molecular scale and promote the development of polymer membranes. In this regard, defining a buffer zone becomes crucial, serving as a region where data from FFM simulations are processed, averaged, and subsequently transferred to the CB model. This ensures a smoother transition of data between the two scales, which is crucial for accurately predicting important properties such as membrane water flux and retention rate [118]. Ensuring data consistency across the microscopic and macroscopic scales is a pivotal aspect of this integration, as shown in Fig. 4a. This involves employing suitable averaging techniques to transform microscopic data into macroscopic parameters, alongside ensuring that the molecular dynamics simulations faithfully represent the chemical and physical properties of the polymer membrane. Techniques such as coarse-graining are invaluable in this context, as they aid in reducing the FFM data resolution to a level that is compatible with the CB model, while still capturing the system's essential behaviors [121, 124]. Synchronization between the FFM simulations and CB models is also a critical consideration, given that FFM simulations operate on a shorter time scale and at a finer spatial resolution compared to CB models. Implementing algorithms to manage these discrepancies is essential, ensuring that the data exchange between the two scales is accurate, consistent, and appropriately timed, thereby providing reliable inputs for both models and enhancing the predictive accuracy of the hybrid FFM/CB approach [126].

#### Algorithms and techniques for model consistency and accuracy

To guarantee accurate and consistent results from the combined FFM simulations and continuum-based (CB) models in the context of polymer-based reverse-osmosis/nanofiltration membranes, it is crucial to develop a proper combination of various methods specialized at different scales in a multiscale scheme. Coarse-graining stands out as a primary technique, where the model used in FFM simulations represents groups of atoms or molecules as singular entities, significantly reducing the computational demand while preserving the essential behaviors of the polymer membrane for more efficient data interaction with the CB model [142], as shown in Fig. 4b. Additionally, the implementation of adaptive resolution schemes plays a vital role in managing the transition between the high-resolution details of the FFM model and the lower-resolution CB model, adjusting the level of detail dynamically based on the specific region of interest. Adaptive resolution simulation typically divides the domain into all-atomistic region and coarse-grained regions and connects them using transition regions. This ensures that critical areas, such as the polymer–solvent interface, receive the necessary attention and modeling accuracy, while less crucial regions can be represented more coarsely, optimizing computational resources [142, 143].

Furthermore, the data transfer process from FFM simulation to CB entails the use of sophisticated data interpolation algorithms, ensuring a seamless translation of high-resolution FFM data into a format that is compatible and accurately represented within the lower-resolution CB framework [144, 145]. To offer a more accurate portrayal of the physical process of solute traversing the membrane, the algorithms of grand canonical molecular dynamics (GCMD) and NEMD can be introduced. The GCMD algorithm is a hybrid MD-Monte Carlo technique, which was used to maintain constant temperature and chemical potential in virtual experiments. This method ensures that the number of particles and system energy vary according to the standard large regular probability distribution [146]. NEMD can be implemented to assess the transport properties of fluids confined in narrow pores [147–150]. The above algorithms and techniques will provide reliable and precise insights for the development and optimization of polymer-based reverse-osmosis/nanofiltration membranes [151].

#### Addressing challenges related to information exchange

Addressing the challenges associated with information exchange between FFM simulations and CB models is crucial when dealing with polymer-based reverse-osmosis/nanofiltration membranes, aiming for precise and reliable predictions. The FFM simulations generate extensive datasets, detailing every atomic or molecular interaction within the system. To integrate information obtained from finer scales into coarser scales in a hierarchical manner, it is necessary to implement of robust and efficient algorithms capable of managing these large volumes of data. These algorithms must extract pertinent information and condense it aptly, preparing it for integration into the CB model, which operates at a significantly coarser resolution [125, 151]. MD simulations mentioned earlier are currently unable to predict the transport characteristics of fluids in graded porous materials composed of thousands of pores or in complex pore networks. An essential aspect of this integration process is the assurance of physical relevance in the parameters being transferred from the FFM simulations to the CB model. This demands meticulous calibration of the FFM model, as well as comprehensive validation against experimental data to confirm that the parameters utilized in the CB model genuinely capture and reflect the physical behavior of the polymer membrane [126, 152]. Addressing these challenges head-on ensures that the integrated FFM/CB model yields reliable, physically accurate predictions, ultimately enhancing our understanding and optimization capabilities regarding polymer-based reverse-osmosis/nanofiltration membranes.

### Multi-technics to get a multiscale picture

#### Integration of continuum-based methods, molecular simulations, and statistical mechanics

In the first layer of this integrative approach, continuum-based methods and molecular simulations offer complementary insights. This large-scale perspective is crucial for practical applications and for understanding the overall behavior of the membrane [153–155]. On the other hand, molecular simulations dive deep into the microscopic world, unraveling the ways in which individual polymer chains, solvent molecules, and solutes interact, as well as how the nanoscale structure of the membrane influences its permeability and selectivity. This level of detail is vital for decoding the mechanisms behind observed phenomena and for pinpointing strategies to optimize membrane performance [156], as shown in Fig. 4c. Xu et al. [157] devised a novel mathematical model for mass transfer that used the tandem resistance model and Monte Carlo simulation methods, then assessed the qualitative and quantitative impacts of parameters such as intrinsic permeability of nanoparticles (NPs), NPs-polymer interlayer, and NPs geometrical parameters on membrane performance. Jiang et al. [158] demonstrated the occurrence of nanoscale molecular transport within CCG membranes through a combination of continuum modelling and MD simulations. They were able to fully characterize the microstructure of the graphene hydrogel membranes and provided valuable insights into the extraordinary electrodynamic nanoflows within the membrane.

Bridging the gap between these scales is the role of statistical mechanics, which serves as a vital intermediary. By deriving macroscopic properties from the behavior of individual particles, statistical mechanics are used to construct or enhance macro-level models through the development of microphysical models, accounting for intermolecular interactions, atomic arrangements, electronic structures, and other relevant factors. It performs micro-simulations, statistically analyzing data they generate to obtain valid information, converting the micro-information into macro-observable physical quantities, validating the accuracy of the models by comparing the predictions provided with the experimental data, and finally integrating the micro- and macro-models into a multiscale modelling framework, to ensure a seamless transition between the microscopic and macroscopic realms, enhancing the coherence and consistency of multiscale models [159]. This integration not only boosts the accuracy and reliability of the models but also opens doors for optimization and innovation. It lays a solid foundation for validating continuum-based models using microscopic data, ensuring that the models accurately reflect the fundamental behaviors of the system. Moreover, it empowers researchers and engineers to identify molecular-level modifications that could lead to improved membrane performance, fostering the development of advanced membranes with superior efficiency and selectivity.

#### Advancements through data-driven modeling and machine learning

Traditional analytical methodologies frequently rely on process-based models. Such models use a mix of mathematical equations based on the process, and they are always somewhat inefficient, less precise, and time-consuming. However, the evolution of polymer-based membrane technology has been significantly fueled by the incorporation of data-driven modeling and machine learning (ML) within multiscale modeling frameworks. The fusion of ML algorithms like the Deep Material Network (DMN) and XGBoost with multiscale modeling has demonstrated a potent capability to decode the complex datasets derived from molecular simulations and experimental investigations, thus overcoming the constraints inherent in traditional analytical methodologies [160, 161]. For instance, predictive modeling enabled by ML has proved to be pivotal in assessing micropollutant removal efficiencies and the performance of thin film nanocomposite membranes in organic solvent nanofiltration scenarios, providing a deeper comprehension of liquid transport dynamics through polymer membranes [162]. Ma et al. [163] adopted the integrated model of Random Forest and XGBoost for training and obtained the optimal model based on hyperparameter optimization, so they highlighted key characteristics affecting the water/salt selectivity of polyamide nanofiltration membranes and proved the feasibility of machine learning method in exploring the complex separation mechanism of NF membranes, as shown in Fig. 4d. Ma et al. used two tree-based models, Random Forest and XGBoost to train the data with an 80:20 ratio of training set to test set in order to optimize the hyper-parameters to obtain the best model. The models were then used to investigate the correlation between the structural parameters (membrane pore radius and zeta potential), experimental conditions (pressure and concentration), and the retention performance of the four salts.

Moreover, combining Data-Driven Modeling and Machine Learning with multiscale modeling is pioneering a trajectory towards more precise and insightful analyses, bridging the microscopic and macroscopic domains of membrane functionality. A significant impact of this convergence is observed in membrane design and discovery, where ML substantially influences the research and development of new materials for energy and environmental applications [164]. The multidimensional paradigm of polymeric membrane design, facilitated by machine learning, notably Bayesian optimization, is revolutionizing the exploration and optimization of membrane materials and fabrication conditions, reducing the dependency on exhaustive trial-and-error experimentation. Through these ML-enhanced frameworks, the iterative cycle of design, testing, and refinement is accelerated, catalyzing rapid advancements in membrane technology and thereby, steering the field towards optimized membrane performance for critical applications like water treatment [165]. Ritt et al. [166] scrutinized the impact of monovalent anions and membrane specificity on salt transportation by means of machine learning thoroughly, uncovering distinctive phenomenological variations in selectivity that arise from the anions. Giro et al. [167] extended the quantitative structure–property relationship methodology using the SMILES training dataset. Additionally, they developed an inverse material design (IMD) engine for machine learning modelling. This work has led to the automated discovery and physical verification of polymer films through end-to-end computational processes.

From a technical standpoint, machine learning in membrane science primarily functions through the analysis and interpretation of complex data sets. Techniques such as Deep Material Network (DMN) and XGBoost are employed to process extensive experimental and simulation data, enabling the identification of intricate patterns and relationships that are not readily apparent. These algorithms facilitate the prediction of membrane performance under various conditions, aiding in the optimization of membrane composition and structure. Moreover, machine learning tools are instrumental in simulating the molecular dynamics of membranes, offering insights into the fundamental mechanisms of solute transport and rejection. This technical application of ML not only augments the precision of membrane research but is also likely to significantly speed up the discovery and improvement of advanced membrane materials, contributing to the development of more efficient separation processes.

## Perspectives and challenges

In the intricate endeavor of multiscale modeling of polymer-based reverse-osmosis/nanofiltration membranes, one of the paramount challenges lies in maintaining a seamless consistency across scales. Each tier from atomic-level interactions to macroscopic functionality must be accurately represented and aligned with its counterparts to ensure that the emergent model faithfully predicts real-world behavior [168]. This integration mandates not only a rigorous calibration and validation of the individual models but also necessitates their adaptation to operate harmoniously within a unified framework. The algorithms must be finely tuned so that the data flow remains robust and the translations of properties between scales preserve physical relevance. This level of precision requires a validation process that is both meticulous and expensive, often encompassing comparisons against experimental data and ensuring that the model predictions align with the known performance characteristics of the membranes.

Concurrently, the computational demands of such sophisticated modeling are formidable. High-fidelity molecular simulations, along with the processing of massive datasets by machine learning algorithms, consume significant computational resources. Effective management of these resources is critical, involving strategic choices about the level of detail necessary at each scale and the allocation of computational effort. For instance, judicious coarse-graining can reduce the resolution of molecular models where detailed interactions are less critical, thereby saving computational power for areas requiring finer scrutiny. Moreover, the integrated modeling approach must navigate the inherent limitations that arise from the synthesis of diverse methodologies. Challenges such as data scarcity for machine learning training, the accuracy of coarse-grained models, and the extrapolation of simulation results to experimental scales must be carefully considered [169–171]. Only through a balanced consideration of these factors can the multiscale approach yield reliable insights, propelling the development of membranes with optimized performance for vital separation and filtration applications. Finally, the choice of a technique is determined by the objective set, whether it is to predict or understand. If the aim is to gain a better comprehension of molecular-scale phenomena to enhance transport and facilitate design, then FFM or CG simulations is the appropriate method. On the other hand, if the objective is to incorporate quick prediction for industry purposes, then CB should be employed. To improve the models used in the latter scenario, integration of data obtained from FFM or CG simulations should be included in CB as well.

Currently, ML is a highly fashionable tool used in three main areas: (i) designing new materials, (ii) predicting properties, and (iii) employing a machine learning-assisted modeling approach to systematically extract accurate coarse-grained representations and force fields for polymer systems. MD simulations are increasingly being integrated with ML to forecast material properties. The molecular configurations derived from MD are characterized by multiple features, such as thermodynamic/transport properties, and serve as the input for ML algorithms. However, for ML to accurately discern input–output patterns, a sufficiently large dataset is required, contingent upon the complexity of the ML model. Generating such a vast dataset from MD simulations (whether involving all atoms or CG models) is not ideal due to their high computational cost. Consequently, new theoretical/numerical ML advancements will be necessary to enable the systematic utilization of ML in predicting transport properties. Nonetheless, describing the membrane at the nanoscale will be essential to comprehensively grasp the microscopic structure governing NF/RO properties.

## Data Availability

No datasets were generated or analysed during the current study.
